# Implementation of a rapid diagnostic assay package for cryptococcosis, histoplasmosis and tuberculosis in people living with HIV in Paraguay

**DOI:** 10.1186/s12879-024-09257-5

**Published:** 2024-04-16

**Authors:** Gloria Aguilar, Gladys Lopez, Omar Sued, Narda Medina, Diego H. Caceres, Jose Pereira, Alexander Jordan, Virgilio Lezcano, Cristina Vicenti, Gustavo Benitez, Tania Samudio, Freddy Perez

**Affiliations:** 1 National HIV Program, Asunción, Paraguay; 2 Facultad de Ciencias de la Salud, Universidad Sudamericana, Amambay, Paraguay; 3 Communicable Diseases Prevention, Control, and Elimination and Environmental Determinants of Health Department, Pan American Health Organization, 525 23rd St. NW, 20037 Washington, DC USA; 4 Mycotic Diseases Branch, Division of Foodborne, Waterborne, and Environmental Diseases (DFWED), ASRT, Inc., Centers for Disease Control and Prevention, 30333 Atlanta, GA USA; 5 IMMY, Inc., 73069 Norman, OK USA; 6 Studies in Translational Microbiology and Emerging Diseases (MICROS) Research Group, School of Medicine and Health Sciences, Universidad del Rosario, Bogota, Colombia; 7 Centro de Especialidades Dermatológicas, Ministerio de Salud Pública y Bienestar Social, San Lorenzo, Paraguay; 8 Mycotic Diseases Branch-Centers for Disease Control and Prevention (CDC), Atlanta, GA USA; 9 Instituto de Medicina Tropical, Asunción, Paraguay; 10 Federal University of Health Sciences of Porto Alegre, Porto Alegre, Brazil

**Keywords:** Opportunistic infections HIV, Diagnostic, Paraguay

## Abstract

**Background:**

Opportunistic infections (OIs) are common causes of mortality among people living with HIV (PLHIV). We determined prevalence and 30-day mortality due to histoplasmosis, cryptococcosis, and TB in PLHIV with advanced HIV disease (AHD).

**Methods:**

PLHIV 18 years and older, with a CD4 + T-cell count of less than 350 cells/mm3 newly diagnosed with HIV infection or re-engaged in care after being without ART for more than 90 days (Group A). The second group included symptomatic PLHIV regardless of ART status or CD4 + T-cell count (Group B); all followed for 30 days. Detection of *Histoplasma* Ag (HisAg) in urine was done by enzyme immunoassay (EIA), *Cryptococcus* antigen (CrAg) was detected in serum and cerebrospinal fluid (CSF) specimens by lateral flow assay (LFA), and lipoarabinomannan (LAM) detection in urine was by LFA (TB LAM) and in sputum by GeneXpert for diagnosis of *Mycobacterium* infections.

**Results:**

From August 2021 to June 2022, 491 PLHIV were enrolled; 482 (98%) had a CD4 + T-cell result, and 381 patients (79%) were classified with AHD according to CD4 + T-cell count (< 200 CD4/mm^3^). Frequency of an OI was 38% (*n* = 145/381). Antigen test positivity rate was 16% (72/467) for TB-LAM, 9% (43/464) for HisAg, and 11% (51/484) for CrAg. Twenty-one of 34 (62%) patients receiving CSF CrAg tests were positive, confirming meningitis. Significant differences in 30-day mortality were observed in patients with an OI (16%) vs. no OI (7%) (*p* = 0.002). Mortality was highest in patients with histoplasmosis (25%), co-infection (22%), cryptococcosis (18% overall; 19% for cryptococcal meningitis), and TB (10%).

**Conclusions:**

TB and fungal OIs, including co-infection, were common in PLHIV in Paraguay and had high associated mortality. Laboratories and health facilities need access to CD4 + T-cell testing and rapid diagnostic assays.

## Introduction

Expanded HIV testing and rapid initiation of antiretroviral therapy (ART) can reduce advanced HIV disease (AHD) which is defined by the World Health Organization (WHO) as those with a CD4 T-cell count below 200 cells/mm^3^ or WHO stage 3 or 4 illness in adults and adolescents [1]. Routine screening for opportunistic infections amongst patients with AHD can increase early detection of individuals who need urgent management to reduce deaths [1]. In Paraguay in 2020, an estimated 16, 233 people were living with HIV (PLHIV). Of these, 89% had been diagnosed, 70% were on ART and 49% were virally suppressed with an undetectable viral load. In 2021, 36% of PLHIV with a recent diagnosis presented AHD [2].

PLHIV with AHD are at higher risk of opportunistic infections (OIs), including cryptococcosis, histoplasmosis, and tuberculosis (TB) [3–6]. The main challenges in identifying these infections are the non-specificity of clinical signs and symptoms, a low clinical suspicion for fungal infections, and limited access to rapid and accurate diagnostic tests [4, 7–9]. Conventional laboratory methods for the diagnosis of these OIs, such as culture or histopathology, present several limitations, including the need for complex laboratory infrastructure, trained professionals, and long turnaround time for results, particularly for diagnosis of histoplasmosis and TB [9–11].

WHO recommends the use of antigen-based tests for the rapid diagnosis of cryptococcosis, histoplasmosis, and TB among PLHIV with AHD [3–6]. The use of LAM is recommended for all individuals with signs and symptoms of TB, as well as for admitted asymptomatic patients with < 200 cell/ mm^3^ or outpatients with < 100 cell4/ mm^3^. WHO also recommends the detection *of Cryptococcus* antigen (CrAg) and *Histoplasma* antigen (HisAg) in individuals with AHD. In Paraguay, national screening of TB in PLHIV has increased from 33% in 2010 to 86% in 2018. In 2020, 8% of all HIV cases registered had a diagnosis of TB [12]. Cryptococcosis and histoplasmosis are endemic mycoses in the region, but the burden of these diseases in Paraguay is unknown.

Previous reports have shown that implementing rapid diagnostics assays (RDA) for OIs can positively impact patient care [9, 13, 14]. The use of these assays has been shown to increase the number of cases diagnosed and reduce mortality [13, 15–17]. Currently, there are commercially available RDAs, which have facilitated the implementation of rapid diagnostics for cryptococcosis, histoplasmosis, and TB. These assays, including enzyme immunoassays (EIA) and lateral flow assays (LFA) support diagnosis of disease through detection of circulating antigen (Ag)Commercial assays are also available for the detection of DNA, including the Biofire® FilmArray® for the diagnosis of cryptococcosis and TB and the Cepheid’s, GeneXpert® Systems for the diagnosis of TB.

This report describes the results from implementation of a package of diagnostic assays for the detection of cryptococcosis, histoplasmosis, and TB in PLHIV at the Instituto de Medicina Tropical in Asuncion, Paraguay. This health facility provides care for 72% of PIHIV enrolled in care at the national level Paraguay. We also describe the prevalence of cryptococcosis, histoplasmosis, and TB, and analyze the 30-day survival of patients with these infections and for all enrolled patients.

## Methods

### Study design and participants

PLHIV were prospectively enrolled at the Instituto de Medicina Tropical in Asuncion, Paraguay from August 2021 to July 2022. Patients were recruited from the emergency room, inpatient ward, and the outpatient HIV clinic. We included adults over the age of 18 years who consented to participate. Two groups were defined, the first included asymptomatic PLHIV with a CD4 + T-cell count of less than 350 cells/mm^3^ newly diagnosed with HIV infection or re-engaged in care after being without ART for more than 90 days (Group A). The second group included symptomatic PLHIV regardless of ART status or CD4 + T-cell count (Group B). Symptomatic PLHIV included those patients with any symptom suggestive of systemic infections within the last 14 days (fever, cough, expectoration, weight loss, night sweats, altered mental status, headache, focal neurological signs, lymphadenopathies, or extensive mucosal or skin lesions).

Individuals were excluded if less than 18 years old, were HIV uninfected or if they had received active treatment during the last 2 weeks for TB and/or systemic fungal diseases.Comparison will be conducted between individuals presenting with OIs and those without, stratified by CD4 T-cell count ranges and examining the distribution of OIs according to CD4 T-cell count.

### Laboratory procedures and case definitions

For asymptomatic individuals, a CD4 + T-cell count was performed if the patient did not have a previous CD4 + T-cell count in the last three months. All patients with respiratory symptoms and sputum production were asked to provide a sputum specimen for GeneXpert MTB/RIF per Paraguay National TB Program guidelines [18].

Microscopic analysis, culture, and molecular testing by GeneXpert MTB/RIF were done on respiratory specimens. Complementary analysis such as the Film Array® (Biomérieux, Marcy-l’Étoile, France) were performed at the attending clinician’s request and in those patients suspected of extra pulmonary infections caused by mycobacteria or others OIs. The clinical management and treatment for any diagnosed OIs were provided by the attending clinician per local practice and national guidelines.

Laboratory assays for cryptococcosis, histoplasmosis, and TB were done following the manufacturer’s instructions. *Cryptococcus* Ag (CrAg) was detected in sera using a commercial LFA kit (CrAg® LFA, IMMY, Norman, OK, US). In patients with positive CrAg, the laboratory requested cerebrospinal fluid (CSF) specimens for CrAg LFA testing to rule out *Cryptococcal* meningitis (CM). India link and CSF fungal culture were also performed on CSF when available. Detection of *Histoplasma* urinary antigen (HGM) was performed using the Clarus *Histoplasma* GM EIA (IMMY, Norman, OK, US), specimens were tested using the standard curve method. A positive result was considered if the specimen presented a concentration of HGM higher or equal to 0.2 ng/ml. For TB, lipoarabinomannan Ag (LAM) was tested in urine (DETERMINE TB LAM Ag, Abbott, Maine, USA). LAM was defined as positive using the grade one cutoff on the manufacturer’s post-2014 reference card.

Cases were defined as follows: (i) *Cryptococcosis*: a positive CrAg in sera. (ii) *Cryptococcal* meningitis: a positive CrAg, Indian ink, or culture in CSF. (iii) *Histoplasmosis*: a positive HGM test or other laboratory evidence of histoplasmosis such as positive histopathology or culture and (iv) Bacteriologically confirmed TB: culture-positive, or a WHO-recommended rapid molecular test such as Xpert MTB/RIF, or a positive TB-LAM test.

### Protocol training, data collection, and monitoring

Prior to patient enrollment, the study staff were trained in appropriate clinical practices, specimen collection, and performing laboratory tests to assure the standard quality of the laboratory procedures. The local study coordinator undertook training sessions with the hospital staff to ensure protocol compliance. Bi-monthly meetings were held to follow protocol progress. A standard case report form was designed using EPI-INFO for data collection of demographic and clinical characteristics, test results and 30-days outcome. Patients unable to be contacted after at least three phone calls or domiciliary visits were considered lost-to-follow-up.

Cross-checks with the national HIV database, the national TB database, and the death registry, were done regularly by the National AIDS Program to evaluate accuracy of study data.

### Ethical approval

The Instituto de Medicina Tropical ethical committee, and the Pan American Health Organization Ethical Research Committee (PAHOERC) approved the protocol under register Pan American Health Organization Ethical Research Committee 0347.01. Signed written consent was sought from each patient before performing any procedure. No personal identifiable information was collected in the database used for the analysis. Data was compiled by trained professionals and stored in a secure database. CDC staff were determined to be non-engaged in the pilot screening activities and therefore the pilot was determined to be exempt from Centers for Disease Control and Prevention (CDC) IRB review.

### Statistical analysis

Statistical Package for the Social Sciences, version 25.0 (SPSS Inc., Chicago, IL, USA), was used for analysis. Categorical variables were summarized as counts and percentages. Continuous variables were summarized using medians and their corresponding interquartile ranges (IQRs). We assessed the association between baseline characteristics and the presence of opportunistic infections at admission using chi-square, fisher’s exact test (categorical variables) and Wilcoxon rank-sum test (continuous variables) [19]. A two-sided *p* value < 0.05 was considered statistically significant.

## Results

### Study population and opportunistic infections

Between August 2021 to June 2022, a total of 491 PLHIV were enrolled for screening based on the inclusion criteria; 365 (74%) were male, 278 (57%) were aged less than 40 years and 58% resided in urban areas (Table 1). At the time of screening, 217 (44%) were newly diagnosed with HIV infection. Three hundred and eighty-one patients (78%) were detected with AHD based on CD4 + T-cell count (< 200 cells/mm3) only.

**Table 1:** Baseline characteristics of the screened patients between August 2021 and June 2022

Characteristics	Total 491 (100%)		With OI 162 (33%)		Without-OI 329 (67%)		P value, x^2^
	n	%	n	%	n	%	
**Sex**							
Male	365	74	127	78	238	72	0.090
Female	126	26	35	22	91	28	
**Age**							
Median [IQR]	37	[30–46]	38	[31–44]	37	[30–46]	0.850
**Residence**							
Rural	158	32	56	35	102	31	0.300
Urban	283	58	86	53	197	60	
Unknown	50	10	20	12	30	9	
Patient group*							
A	68	14	7	4	61	19	< 0.0001
B	423	86	155	96	268	82	
**New HIV diagnosis**							
Yes	217	44	68	42	149	45	0.275
No	274	56	94	58	180	55	
**CD4 + T-cell count**							
< 50	150	31	67	41	83	25	< 0.0001
50–99	119	24	47	29	72	22	
100–199	112	23	31	19	81	25	
200–350	74	15	15	9	59	18	
>350	27	6	0	0.0	27	8	
Unknown^±^	9	2	2	1	7	2	

OI = Opportunistic infection

*Group A: asymptomatic PLHIV with CD4 count < 350 cells/mm3, newly HIV diagnosed or re-engaged in care after being without ART for > 90 days. Group B: symptomatic PLHIV regardless of using ART or CD4 count

^±^Patients without a recent CD4 cell count at the time of the opportunistic infections screening

Out of the 491 patients enrolled, 162 (33%) were diagnosed with at least one of the OIs being screened for. Of these 92 (19%) had a diagnosis of TB, 52 (11%) had cryptococcosis (51 diagnosed by serum LFA-CrAg and one through culture) and 43 (9%) had histoplasmosis. Among the 162 patients with a diagnosed OI, 23 patients (14%) had multiple OIs (histoplasmosis and TB in 16 patients (70%); cryptococcosis and TB in 4 patients (17%); cryptococcosis and histoplasmosis in one (4%); triple infection in two patients (9%) **(**Table 2**).**

**Table 2:** Frequency of opportunistic infection based on CD4 + T-cell count*

Opportunistic infection		CD4 + T-cell count						Unknown±
		Overall	< 50	50–99	100–199	200–350	> 350	
Histoplasmosis	+	43 **(9%)**	20 **(14%)**	13 **(11%)**	4 **(4%)**	5 **(7%)**	0	1 **(13%)**
	Screened	464	144	116	103	68	25	8
Cryptococcosis	+	52 **(11%)**	23 **(16%)**	16 **(14%)**	11 **(10%)**	2 **(3%)**	0	0
	Screened	485	148	117	110	74	27	9
Tuberculosis	+	92 **(19%)**	41 **(28%)**	21 **(18%)**	20 **(19%)**	9 **(13%)**	0	1 **(14.3%)**
	Screened	474	145	117	108	71	26	7

*All cases of cryptococcosis and tuberculosis cases were included regardless of the diagnostic assay. The table does not discriminate multiple infections. ± Patients without a recent CD4 + T-cell count at the time of the opportunistic infections screening

Comparing patients with (*n* = 162) and without any of these three OIs (*n* = 329), the median CD4 + T-cell count was significantly lower in patients with OIs than those without OIs (median CD4 + T-cell count 59 cells/mm^3^ vs. 106 cells/mm^3^ respectively, *p* < 0.0001). In asymptomatic patients with CD4 + T-cell < 350 cells/mm^3^ (group A, *n* = 68), seven patients (10%) were diagnosed with an OI. Of these, one had cryptococcosis without meningitis, two had histoplasmosis, three had TB and one had TB and histoplasmosis co-infection. In contrast, in symptomatic patients (group B, *n* = 423), 155 (37%) were diagnosed with an OI (*p* < 0.0001) **(**Table 1**)**.

**Fig. 1:**
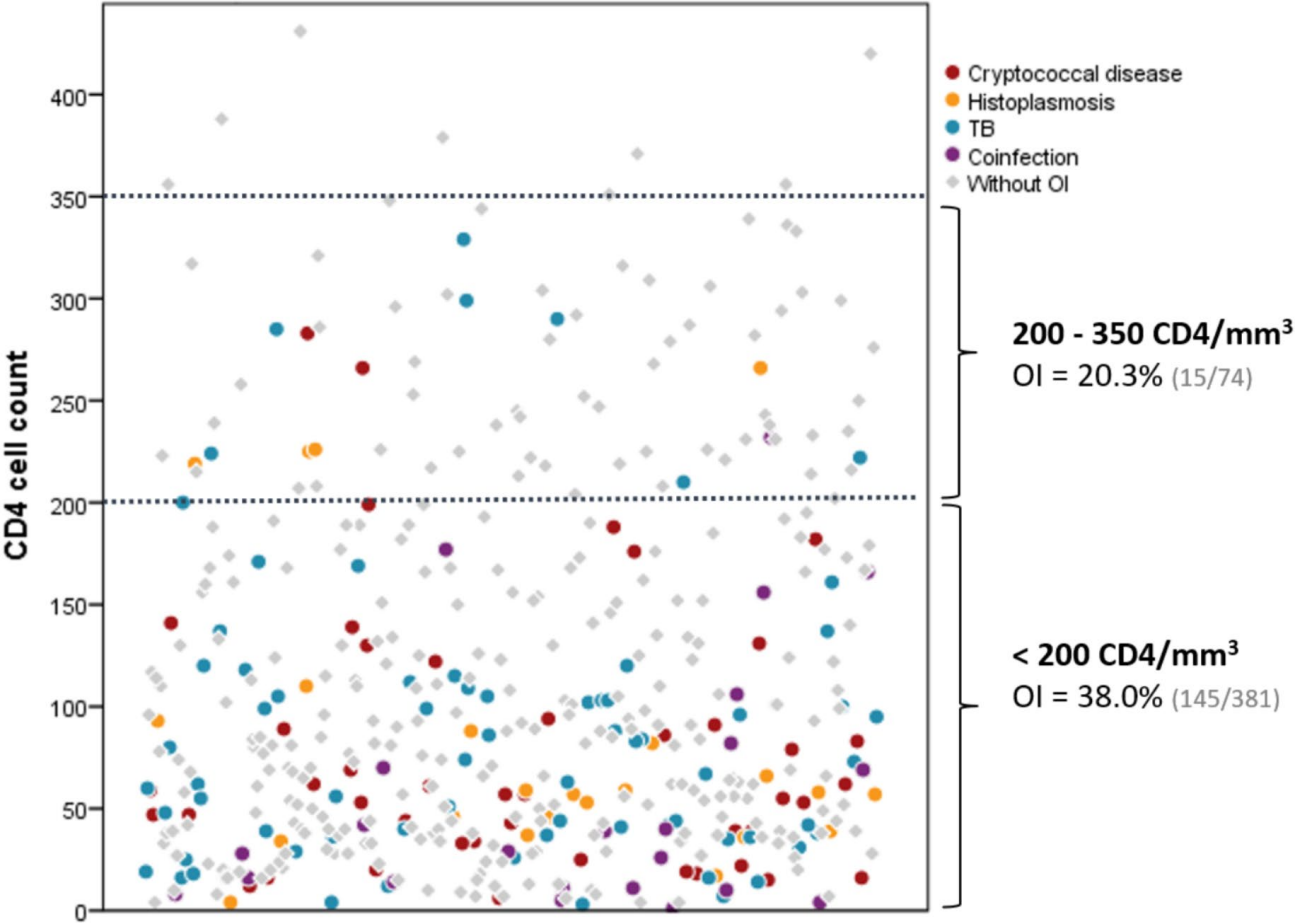
Distribution of opportunistic infections based on CD4 + T-cells count. OI = opportunistic infections; TB = tuberculosis

Among patients with CD4 + T-cells < 200mm^3^, the frequency of the targeted OIs was 38% (*n* = 145). Among patients with CD4 + T-cell counts between 200 and 350/mm^3^, the frequency of OIs was 20% (P = *0.0035*) (Fig. 1). All targeted OIs were found in patients with CD4 + T cells ≤ 350/mm^3^. The frequency of OIs increased as the count of CD4 + Tcells decreased (Table 2). Two cases of OI (one histoplasmosis and one TB ) occurred in patients without a CD4 + T-cell count.

## Opportunistic infection diagnosis

For histoplasmosis screening, 464 of 491 (95%) patients were tested for *Histoplasma* antigen detection in urine (Table 3). Of these, 43 (9%) tested positive. For those not tested, lack of referral of urine to the laboratory for testing was given as the main cause. For *Cryptococcus* screening, 484 of 491 (99%) patients were tested using CrAg LFA in sera, 51 (10%) were positive. One patient, for whom serum CrAg testing was not conducted, was diagnosed with cryptococcal meningitis through culture and Indian ink examination of CSF. Among the 51 patients with positive CrAg in sera, testing of cerebrospinal samples (CSF) was done in 34 patients (67%). Twenty-one of the 34 (62%) CSF samples tested positive for CrAg, confirming meningitis. Detection of CrAg in CSF samples by the LFA-CrAg assay yielded a higher positivity rate than did CSF culture (62% vs. 50%). At the time of screening, 5% of the asymptomatic patients with AHD received a diagnosis of an OI.

**Table 3:** Positivity of the tests used for screening of opportunistic infections

Diagnostic assay	Histoplasmosis (*n* = 43)		
	Tested	+	%
Urine Ag	464	43	9%
Cryptococcosis (n = 52)*			
Serum LFA-CrAg	484	51	11%
CSF LFA-CrAg	34	21	62%
CSF culture	22	11	50%
CSF India ink	23	4	17.4%
**Tuberculosis (n = 92)**			
TB-LAM	467	72	15%
TB GeneXpert	204	35	17%
TB Culture	102	22	22%
TB-LAM + GeneXpert	197	62	31%

Ag = antigen; CSF = cerebrospinal fluid; LFA = lateral flow assay; TB = tuberculosis

*For one patient, culture was done for diagnosis, reason why no CrAg LFA in sera was performed

Of all enrolled patients, 467 (95%) were screened with TB-LAM, 204 (42%) with GeneXpert, and 102 (21%) were tested by culture. Of these, 72 (15%), 35 (17%), and 22 (22%) were positive, respectively. The highest positivity rate was observed in patients receiving both TB-LAM and GeneXpert testing (Table 3). In this group of 197 patients, a total of 62 TB cases were diagnosed. Among them, 29 (47%) were diagnosed solely through TB-LAM, 15 (24%) through GeneXpert alone, and 29% (*n* = 18, 29%) through a combination of both tests.

### Treatment and outcomes

Of the 92 cases diagnosed with TB, 83 (90%) received antituberculosis treatment. Of the 43 histoplasmosis cases, 36 (84%) initiated antifungal treatment, (21 with amphotericin B deoxycholate, 12 with itraconazole, three with fluconazole). For cryptococcosis (*n* = 52), all patients initiated antifungal treatment for either cryptococcal meningitis or non-meningeal cryptococcosis based on confirmation of cryptococcal meningitis and clinical judgment; (31 with amphotericin B and fluconazole, 21 with fluconazole for pre-emptive treatment).

Of all enrolled patients, 486 were followed prospectively for 30-days. Mortality in this period was 10% (49 patients), including 26 (53%) patients with an OI diagnosed in this study (eight cryptococcosis, seven TB, six histoplasmosis, and five multiple opportunistic infections). Patients with OIs had significantly higher mortality than patients without opportunistic infections (16% vs. 7%; *p* = 0.002). The highest mortality rate (25%) was observed in patients with histoplasmosis, followed by multiple opportunistic infections (22%), cryptococcosis (18%), and TB (10%) (*p* = 0.004). For those with confirmed cryptococcal meningitis, the mortality rate was 19%.

## Discussion

**Fig. 2:**
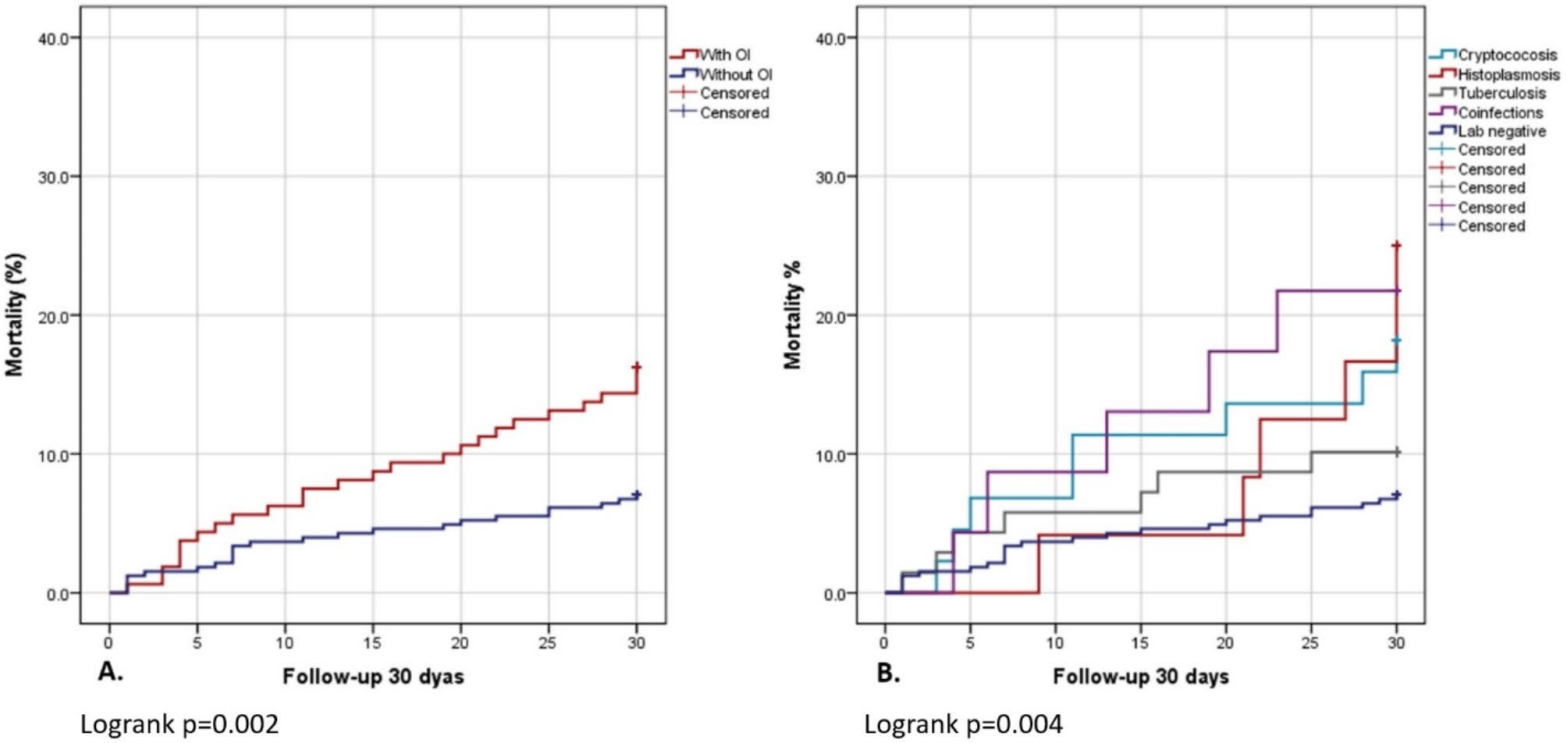
Mortality at 30 days in the cohort of patients screened for opportunistic infection in Paraguay. **A** Patients with and without opportunistic infections; **B** Patients with tuberculosis, cryptococcal disease, histoplasmosis, and with multiple opportunistic infections. Opportunistic infections = OIs

In this prospective study, one out of three PLHIV with advanced HIV or new HIV diagnosis had cryptococcosis, histoplasmosis or TB. Routine screening using rapid diagnostic assays provided early detection of these OIs. Here, we found that most PLHIV enrolled had AHD (79%). As shown in Fig. 2, the OIs evaluated in this study were more frequent in patients with very low CD4 + T-cell counts (< 50 cells/mm^3^); all detected cases of the targeted OIs were in patients with ≤ 350 cells/mm^3^. The WHO recommends screening for CrAg in patients with < 200 cells/mm^3^ and for TB LAM < 100 cells/mm^3^ among outpatients as well as any symptomatic inpatients [6, 20]. Our findings align with results from a recent analysis in Guatemala, in which 95% of OIs were diagnosed using a 350 CD4 + T-cell threshold in a screening program for newly HIV diagnosed patients [13]. When considering an appropriate CD4 + T-cell count threshold for implementing this diagnostic package for opportunistic infections, program planners must balance the burden of AHD and of the targeted OIs, while also considering the cost implications for HIV programs. A study in 2023 by Rajasingham and colleges assessed the cost-effectiveness of integrating Histoplasma antigen testing in patients with AHD at a large scale, and estimated that it is cost-effective [21]. However, the public health impact, and cost of an approach that targets individuals with CD4 + T-cell counts between 200 and 350 for routine screening with these rapid OI diagnostics requires additional evaluation and should be weighed by program planners in the context of the local HIV and OI burden and resource availability [13]. 

Data on the burden of these diseases in Paraguay is historically scarce. The overall frequency of CrAg in serum samples in the present study (11%) is similar to that reported in Brazil with a frequency of 11.2% in patients with AHD [22]. Several studies have reported the diagnostic value of the LFA in detecting cryptococcosis, including in asymptomatic patients. *Cryptococcus* antigenemia is a well described risk factor for developing cryptococcal meningitis, and requires antifungal therapy tailored to whether concurrent CM is confirmed or suspected [14, 23]. Here, we found that 62% of the serum CrAg positive cases had CM. Within 30 days of follow up 19% of serum CrAg positive patients had died, a result which emphasizes the need for routine and rapid access to appropriate antifungal treatment for patients with cryptococcosis.

Histoplasmosis imposes a significant burden in this region, with prevalences of 10% and higher reported among individuals with AHD in the region [24, 25]. Co-occurrence with TB and other opportunistic infections is common, highlighting the need for improved capacity to detect histoplasmosis as part of the routine workup of individuals with AHD in this region [26, 27].

Our screening strategy classified patients based on the presence or absence of general symptoms, which is a key clinical indicator. At the time of screening, 5% of the asymptomatic patients with AHD received a diagnosis of an OI. In the absence of this strategy, these cases may have progressed to severe disease, requiring more complex care with a higher likelihood of negative outcomes.

Using Histoplasma Ag testing, a total of 43 people with histoplasmosis were detected. It is important to highlight that histoplasmosis was the only OI in 24 patients, in the remaining 19 (44% patients), coinfection with histoplasmosis and other OIs occurred, TB coinfection being the most common (18 of 19 co-infected). This coinfection is commonly reported in Latin America. Several case series and cohorts of PLHIV report the co-occurrence of histoplasmosis-TB ranging from 2 to 38% [11]. A recent review of studies in Latin America which used *Histoplasma* Ag testing showed that use of the test had a positive impact on case detection, increased diagnostic yield, and in some reports doubled the number of diagnosed cases [28, 29]. Additionally, a reduction in mortality of patients diagnosed with histoplasmosis was reported when using rapid antigen testing [30].

Our implementation study used urine antigen testing to detect histoplasmosis, and a small number of cases could have been missed due to challenges in collection of urine, or due to problems in the transport of urine specimens to the laboratory. The package of diagnosis provided here could be further improved by the incorporation of point of care LFA for histoplasmosis.

There is limited data on how to optimally integrate different assays for TB in the region. In this study, when Xpert® MTB/RIF Ultra - Cepheid and TB-LAM tests were both used, the urine TB-LAM test had an incremental yield of 47%. The combination of both assays identified a higher proportion of TB cases (62 of 197 patients tested). These findings are consistent with a multicenter study in which the combination of TB-LAM and GeneXpert tests had the highest sensitivity [31]. Thus, our data supports the importance of incorporating both tests into routine health care for PLHIV. Additional studies including qualitative methods are needed to identify the challenges and gaps in integrating these tests in the package of care for PLHIV.

Overall, the 30-day mortality in this cohort was high and, as expected, was higher among patients with opportunistic infections. Patients with histoplasmosis and those with multiple OIs had the highest mortality rates of 25% and 22% respectively. Most of these patients were highly immunocompromised, with the median CD4+ -cell counts the lowest among patients with multiple OIs (median 42 cells/mm^3^). The most frequent common co-infection was histoplasmosis and TB (70%). Treatment of histoplasmosis/TB coinfection is challenging due to the pharmacokinetic drug-drug interactions of itraconazole and some common TB medicines [8]. Concerning histoplasmosis, clinical manifestations are nonspecific and often resemble those produced by other common infections such as TB. In the absence of a strategy to accurately detect both infections, one of these diagnoses can easily be missed or delayed.

Interestingly, patients with TB presented with the lowest mortality rate in this study, though theTB mortality of 10% found in this study was still high. The implementation of a package of diagnosis such as the one provided in this study promotes rapid start of ART while also allowing for appropriate delay of ART initiation in certain situations (e.g. if cryptococcal meningitis is detected in ART-naive patients), as is recommended by the WHO guidelines for managing AHD [3].

Our study has several limitations. It was not designed to directly evaluate the impact of these interventions on patient mortality. However, we were able to document a high mortality at 30-day follow up. Longer patient follow-up might be helpful to identify the long-term outcomes after the introduction of these tests in addition to analyzing the causes of mortality among HIV patients, especially those due to specific opportunistic infections, including TB diagnosed without bacteriological confirmation [32]. Second, some samples were not collected or processed due to errors during sampling procedures, inability of the patient to provide urine, or samples not taken to the laboratory. Nevertheless, almost 95% of the individuals provided urine samples and 99% provided blood for testing. Treatment regimens were heterogenic in this study, with challenges related to access to fluconazole, lipid formulation of amphotericin liposomal, amphotericin B deoxycholate and 5-flucytosine. This could have had a negative impact on the outcomes of patients with histoplasmosis and cryptococcosis. Finally, the study findings apply to the Paraguayan setting, where HIV care is largely centralized in one hospital and the results are influenced by the local epidemiology. Therefore, findings might not be directly generalizable to other Latin American contexts. PAHO is supporting similar studies in other countries to evaluate similar approaches in other settings.

## Conclusions

This study demonstrates the added value of the inclusion of rapid screening with antigen-based tests for the diagnosis of cryptococcosis, histoplasmosis, and TB among HIV individuals with advanced disease who are initiating or returning to care in Paraguay. The intervention was feasible and led to the identification of a large number of patients with OIs, the prevalence of which was associated with lower CD4 + T-cell counts and higher mortality. All cases of OIs occurred in individuals with CD4 + T-cell counts below 350 CD4/mm^3^. Patients with AHD were significantly more likely to have OIs than those with CD4 + T-cell count between 200 and 350. However, a substantial proportion (20%) of patients with CD4 + T-cell counts between 200 and 350 were detected with OIs. Determining the right CD4 + T-cell count threshold for integrating a diagnostic package for opportunistic infections requires a detailed assessment of the local burden of AHD, the frequency of OIs being targeted, and the cost implications and cost-effectiveness for the HIV program. There is a need to ensure the availability of RDAs and the access to treatment for these OIs to reduce HIV-associated mortality. The results of this demonstration project provide valuable evidence which may inform optimization of national policies and updates of national guidelines for PLHIV with advanced HIV.

## Data Availability

Data is provided within the manuscript.
